# Global Introduction of New Multidrug-Resistant Tuberculosis Drugs—Balancing Regulation with Urgent Patient Needs

**DOI:** 10.3201/eid2203.151228

**Published:** 2016-03

**Authors:** Timothy Sullivan, Yanis Ben Amor

**Affiliations:** Icahn School of Medicine at Mount Sinai, New York, New York, USA (T. Sullivan);; The Earth Institute at Columbia University, New York (Y. Ben Amor)

**Keywords:** tuberculosis and other mycobacteria, antimicrobial resistance, multidrug-resistant tuberculosis, MDR TB, MDR TB drugs, global introduction, extensively drug resistant TB, XDR TB, bacteria

## Abstract

New treatments for multidrug-resistant tuberculosis (MDR TB) are urgently needed. Two new drugs, bedaquiline and delamanid, have recently been released, and several new drugs and treatment regimens are in the pipeline. Misuse of TB drugs is a principal cause of drug resistance. As new drugs and regimens reach the market, the need to make them available to patients must be balanced with regulation of their use so that resistance to the new drugs can be prevented. To foster the rational use of new drugs, we propose 1) expanding/strengthening the capacity for drug susceptibility testing, beginning with countries with a high TB burden; 2) regulating prescribing practices by banning over-the-counter sale of TB drugs and enacting an accreditation system whereby providers must be certified to prescribe new drugs; and 3) decentralizing MDR TB care in rural communities by employing trained community health workers, using promising mobile technologies, and enlisting the aid of civil society organizations.

The World Health Organization (WHO) recently described the global effects of multidrug-resistant tuberculosis (MDR TB) as a “public health crisis” (*1*). In 2013, an estimated 480,000 new cases and 210,000 deaths were caused by MDR TB, which is defined by resistance to at least isoniazid and rifampin, the 2 most effective anti-TB drugs (*1*). In addition, ≈9% of MDR TB cases are believed to be extensively drug-resistant (XDR TB), which implies additional resistance to any fluoroquinolone and to at least 1 injectable second-line drug (*1*). Treatment of MDR and XDR TB require prolonged therapy with toxic, poorly tolerated medications, and for patients in developing countries, access to active drugs may be limited. As a result, outcomes for MDR and XDR TB treatment have been discouraging: in a 2011 cohort, treatment was successful for only 48% of patients with MDR TB and for 22% of those with XDR TB (*1*).

Fortunately, 2 drugs active against drug-resistant TB, bedaquiline and delamanid, have recently been approved (delamanid by the European Medicines Agency and bedaquiline by the US Food and Drug Administration and the European Medicines Agency) (*2*,*3*), and several others are being developed (*4*). Because TB drugs need to be given in combination to prevent drug resistance, trials are underway to design treatment regimens that include using bedaquiline and delamanid for MDR TB (*5*). The new drugs and regimens offer the hope of additional effective MDR and XDR TB treatment options for patients who urgently need them. However, given the propensity of *Mycobacterium tuberculosis* to rapidly develop resistance to antibiotics, the worldwide release of new therapies also raises critical questions regarding the need for drug restrictions. In 2014 the WHO published a policy implementation package that described conditions necessary for rapidly introducing new TB drugs while maintaining patient safety and treatment efficacy (*6*). Similarly, in 2015 the European Centre for Disease Prevention and Control released recommendations for the responsible use of new TB drugs in European countries, which included several preconditions that should be in place at the national level before new TB drugs are used (*7*). These actions by WHO and the European Centre for Disease Prevention and Control highlight a fundamental dilemma regarding the use of new TB drugs: as new drugs are released, how can the urgent need to make them widely available be balanced with the need to regulate their use, so that drug resistance can be prevented and the promise of novel therapies is not lost?

## Argument for Restricting New Drugs

Successful TB treatment, for drug-resistant or non–drug-resistant TB, requires the use of multiple active drugs, and one particularly troubling cause of treatment failure is the prescription of inadequate or inappropriate drug regimens. For example, a 2010 survey of 106 private practitioners in Mumbai, India, found that only 6 prescribed appropriate TB treatment and that the group prescribed 63 different regimens (*8*). Similarly, another study reported that 89.3% of private physicians surveyed in the Philippines usually treated TB with inappropriate regimens (*9*). Medication errors may also occur within public clinics: a 2002 study found that dosing errors were common within the national TB programs of Kenya, Nepal, and Senegal (*10*). In many areas, anti-TB medications are available over-the-counter with no input from physicians at all (*11*).

Exposure to inadequate treatment is one of the principal causes of TB drug resistance. Antimicrobial drug resistance, including resistance to TB drugs, has been acknowledged by WHO as a serious threat to global public health, and this resistance is thought to be driven in part by inadequate regulation of prescribers and the irrational use of antibiotics (*12*). Given the evidence of poor prescribing practices worldwide, some may argue that restricting the use of new TB drugs to referral hospitals, public clinics, or specially credentialed providers may help promote correct prescribing and therefore prevent the development of resistance. Some countries have already enacted such restrictions on TB treatment to eliminate improper prescribing practices. In Brazil, for example, TB is treated exclusively within the public health system, and most TB drugs are only available through government pharmacies (*13*). Despite the country’s identification as one of the 22 high TB burden countries worldwide, incidence of MDR TB in Brazil has remained relatively low (*1*). Such restrictions are absent from many other countries with a high TB burden. For example, in 2014, Médecins Sans Frontières called for urgent regulation of the private TB drug market in India, to help slow the rising rates of TB drug resistance there (*14*).

In addition to appropriate prescribing practices, successful treatment of drug-resistant TB is aided by timely and accurate drug-susceptibility testing (DST) of the agents being used. Although standardized DST for the newest drugs is still in development, for other components of MDR TB treatment regimens, DST is more established. However, access to DST for second-line drugs is severely limited in many countries (*15*), and where it is available, it may only be performed by national reference laboratories or at large referral centers. Therefore, limiting the use of new drugs to these centers might help ensure their appropriate use as part of effective regimens.

Another potential benefit of restricting the use of new drugs to specialty centers or highly trained providers would be to allow closer monitoring of adverse drug reactions and interactions. Although the side effects of established TB therapies are well known, some new anti-TB medications have distinct toxicity profiles that may not be fully recognized by inexperienced prescribers. Additionally, some toxicities and interactions of new drugs may not yet be known, and therefore patients may benefit from more thorough monitoring for unexpected adverse reactions. WHO has recommended that all centers where bedaquiline or delamanid are prescribed have programs for preventing, detecting, and managing adverse drug reactions and interactions that may be unique to these medications (*16*,*17*).

## Dangers of Restricting New Drugs

Given the extraordinary effects of disease and death caused by MDR and XDR TB, any effort to restrict new drugs must be balanced with the need to rapidly distribute potentially life-saving therapies where they are needed. Recent reports of bedaquiline compassionate use programs suggest that the drug can be safe and effective when combined with other active drugs to treat MDR and XDR TB (*18*,*19*). WHO has identified the scale-up of MDR TB treatment in high-TB-burden countries as a major priority, and restricting the use of new drugs may disrupt global efforts to expand the delivery of much-needed MDR TB care.

The effects of drug regulation on treatment scale-up were seen after the Green Light Committee (GLC) was established in 2000. The GLC was created by the Stop TB Partnership to ensure that low-cost, quality-assured second-line TB drugs reached approved treatment programs. The committee verified that programs adhered to WHO guidelines and procured all drugs used to treat MDR TB by programs funded by the Global Fund to Fight AIDS, Tuberculosis and Malaria.

Although the GLC successfully obtained drugs and verified their appropriate use, after 10 years only 29,000 MDR TB patients had been started on treatment through this mechanism (*20*), a small fraction of cases worldwide. The committee members recognized that to meet the global need for MDR TB treatment, they needed to reconsider their approach to regulation (*20*). The GLC has since been repurposed into a less centralized program focused on the rapid scale-up of care and greater availability of drugs (*21*). The experience of the GLC seems to illustrate the following observation, however. Because GLC provided assistance and training to improve the program quality in countries that did not meet eligibility criteria, the limited number of MDR TB patients who began appropriate treatment through this mechanism may also have reflected countries’ inability to import specific second-line drugs.

Restricting new drugs to referral centers, another seemingly beneficial regulation, may also obstruct access to care. If novel therapies for MDR TB are only available in large hospitals, they will not be accessible to patients in rural areas. Studies in Ethiopia, Malaysia, and Argentina, for example, have linked poor TB treatment adherence with transportation costs or the distance needed to travel to receive care (*22*–*24*). Although it was previously thought that MDR TB could be successfully treated only in referral hospitals, community-based outpatient therapy has also been effective (*25*–*27*). Presumably, new drugs or regimens could be incorporated into existing community-based MDR TB treatment programs with adequate training and oversight. Barriers to access can be devastating to persons with highly resistant disease. Countries have reported multiple problems related to restricting access, such as long waiting lists, patients who relocated to gain access to treatment being lost to follow up when they needed to return home, and the exclusion of vulnerable populations such as refugees, prisoners, and migrant populations (*28*).

In addition, although a principal argument for restricting new TB drugs is to limit the development of resistance by limiting their misuse, recent data suggest that transmitted drug resistance, as opposed to treatment failure, may be an increasing cause of drug-resistant TB cases (*29*). Providing effective treatment with new drugs may therefore actually help reduce resistance rates by curing patients of MDR TB and reducing their infectiousness.

## The Way Forward: Regulation without Obstruction

To meet the worldwide demand for new MDR TB therapies, while also limiting drug resistance and monitoring for adverse effects, the public health community needs an approach to treatment regulation that does not hinder care. Efforts should thus focus on improving DST, ensuring appropriate prescribing practices, and promoting effective community-based MDR TB treatment (Table). 

**Table:** Suggested plan for ensuring the appropriate use of new TB drugs and regimens*

Goals	Support	Feasibility/precedent
1. Improve DST in high-TB-burden countries		
A. Increase number of laboratories performing TB culture and DST	Key goal of WHO (*1*). Funding available from UNITAID, FIND, GLI, Global Drug Facility, Global Fund, United States government, World Bank	Ongoing global scale-up of DST during 2006–2015. EXPAND-TB project has improved technology in 97 TB laboratories worldwide (*1*)
B. Improve TB diagnostic technology in existing laboratories, including rollout of molecular diagnostics	WHO, NTPs	>3,000 GeneXpert machines procured at concessional prices since WHO recommended use in 2010 (*1*)
C. Develop a specimen bank of TB strains resistant to new antibiotics	WHO, private sector (both diagnostic and pharmaceutical companies)	TDR TB strain bank launched by Special Program for Research and Training in Tropical Diseases
2. Improve prescribing practices in high TB burden countries		
A. Establish accreditation process for prescribers of new TB drugs	National governments, NTPs, pharmaceutical companies	Similar programs instituted for laboratory services in low-resource settings (*25*–*27*)
B. Ban over-the-counter sale of TB drugs	Widely supported by many NGOs and other authorities (*15*)	Common practice in many countries; new regulations instituted in India in 2014
3. Support community-based treatment of MDR TB		
A. Employ community health workers to assist with MDR TB treatment	Supported by WHO (*1*); to be implemented by local NTPs and NGOs	Beneficial role of CHWs in TB care well described (*1*)
B. Use emerging mobile technologies to monitor for adherence and adverse effects	WHO recently acknowledged value of mobile health technology (*35*)	Well-studied for HIV care; data for use in TB management emerging (*29*–*34*)
C. Enlist support of existing civil society organizations	Wide support on global, national, and local level	Many such organizations already invested in improving TB care

*TB, tuberculosis; DST, drug-susceptibility testing; WHO, World Health Organization; FIND, Foundation for Innovative New Diagnostics; GLI, Global Laboratory Initiative; EXPAND-TB, Expanding Access to New Diagnostics for TB; NTPs, national TB programs; TDR TB, totally drug-resistant TB; MDR TB, multidrug-resistant TB; NGOs, nongovernmental organizations; CHWs, community health workers.

First, the availability and reliability of DST in developing countries must be improved to ensure that new drugs are used appropriately as part of effective multidrug regimens. Strengthening laboratory support in countries with a high TB burden has already been identified as a key goal by WHO and the Stop TB Partnership (*1*). 

WHO’s 2014 Global TB Report described several recent improvements in worldwide DST standards, including the rapid global rollout of Xpert MTB/RIF systems and the Expanding Access to New Diagnostics for TB (EXPAND-TB) Project, a collaboration to improve TB laboratory support in 27 countries that contain ≈40% of global MDR TB cases (*1*). However, that report also noted that in 2013, 12 of 27 countries with a high burden of MDR TB did not yet have an adequate number of laboratories that performed TB culture and DST, and that quality assurance in many laboratories is lacking. Therefore, despite recent gains, intensive laboratory strengthening is still needed. Also, in countries in which new TB drugs are introduced, WHO and national TB programs (NTPs) must closely monitor all patients receiving new drugs for the development of resistance. Not only will this monitoring help limit the spread of new drug-resistant strains, but it will also make it possible to collect a bank of specimens for researchers and diagnostic companies to identify emerging resistance mutations and develop relevant diagnostic tests (DST for the new drugs). These tests could eventually be introduced to help guide the use of new drugs and aid in the introduction of additional compounds still undergoing development.

Second, in countries such as India where TB is frequently treated in private clinics, providers should be better trained in MDR TB treatment, and, if possible, prescribing privileges should be limited only to trained prescribers. Although Brazil has shown that shifting treatment to the public sector has led to more stringent TB treatment oversight, private practitioners in India are still major providers of TB care and excluding them from TB treatment may not be feasible. Efforts are ongoing within the Indian national TB control program to better engage the private sector and to provide incentives to doctors to deliver appropriate TB care (*30*,*31*).

For countries in which a large proportion of private sector providers are involved in TB management, we strongly recommend the introduction of an accreditation mechanism developed by local NTPs, with guidance from the WHO. The accreditation process would not only verify providers’ knowledge of current treatment guidelines, it would also educate them on the rational use of new drugs and correct regimens and teach them to recognize and treat possible adverse effects of newly available medications. It would also ensure that the private sector participates in the pharmacovigilance process for the new drugs. Furthermore, an accreditation system could also be used to help foster greater oversight of MDR TB care, including increasing enrollment of MDR TB patients in national registries, encouraging more thorough contact tracing by using NTP and public sector channels once patients are identified and registered by accredited physicians, and improving tracking of adverse events related to new drugs. There are several examples of accreditation programs that were successfully introduced in low-resource settings, and had marked public health effects (*32*,*33*), but involvement of the national government and availability of donor funds are critical (*34*).

The accreditation process should be made mandatory and free to ensure a large national coverage and impact. Even if accreditation were not mandatory, private practitioners would presumably be given incentives to become accredited because accreditation would provide them with a uniquely developed qualification, attesting to their competency and easily identifiable by TB patients. In parallel, local NTPs and civil society organizations should develop campaigns to promote this accreditation to patients and to urge them to only visit accredited practitioners. Such a system would foster the safe use of new compounds, eliminate the prescription of new drugs by unqualified practitioners, and also strengthen overall management of TB in these countries. Because antibiotics are available without prescriptions in many countries, assuring the use of appropriate treatment regimens would also require a ban on the over-the-counter sale of new TB drugs (*35*). In March 2014, the government of India instituted new prescription restrictions on 46 medications, including all first-line anti-TB drugs. The effect of this legislation on prescribing and treatment practices is not yet known.

Third, in rural areas, the management of TB and MDR TB patients should be decentralized to the community level. This can be achieved by using paid community health workers (CHWs) for community-based management of TB and MDR TB, harnessing new technologies to promote appropriate use of drugs, and enlisting support of existing civil society organizations.

CHWs can be trained to monitor patients for treatment compliance, administer injectable second-line drugs, and refer cases with a poor treatment response or severe medication side effects to a higher level of care. CHWs can also utilize new technologies, such as mobile devices, to monitor compliance (*36*), to collect patient data, and to communicate questions or concerns with providers. In addition, these devices can be distributed directly to patients to issue treatment reminders, monitor adherence to therapy, provide educational material related to TB treatment and prevention, and transmit information about adverse events back to prescribers (*37*). If used on a large scale, electronic monitoring with mobile devices might allow detection in real time of previously undescribed adverse drug reactions or interactions. Use of text message communication has been associated with improved adherence to HIV treatment in developing countries (*38*–*40*), and by similarly incorporating these technologies into TB care (*41*), more regulation may be possible without limiting access to treatment. Video-based directly-observed therapy using mobile devices has also recently been shown to be effective (*42*), and other trials evaluating the effects of mobile technology on TB treatment and outcomes are ongoing. Finally, local civil society organizations can be involved to help educate patients regarding TB prevention and treatment, and to reinforce positive messages that TB is curable and that treatment is free. These organizations should be empowered to form support groups within local communities to assist TB and MDR TB patients throughout their treatment.

Bedaquiline, delamanid, and other forthcoming drugs have the potential to greatly improve MDR and XDR TB treatment outcomes worldwide. The appropriate use of these medications along with other active drugs is essential to prevent resistance. Although the release of new drugs and regimens should not prompt the introduction of potentially harmful treatment restrictions, a nuanced use of some restrictions could be introduced without obstructing appropriate treatment. The arrival of promising new drugs should also be seen as an opportunity to strengthen existing TB diagnostic and treatment efforts, so that the expected benefits of new therapies may reach patients quickly and safely.
